# A pancreatic exocrine-like cell regulatory circuit operating in the upper stomach of the sea urchin *Strongylocentrotus purpuratus* larva

**DOI:** 10.1186/s12862-016-0686-0

**Published:** 2016-05-26

**Authors:** Margherita Perillo, Yue Julia Wang, Steven D. Leach, Maria Ina Arnone

**Affiliations:** Biology and Evolution of Marine Organisms, Stazione Zoologica Anton Dohrn, Napoli, 80121 Italy; Department of Surgery and the McKusick Nathans Institute for Genetic Medicine, Johns Hopkins University, Baltimore, MD 21205 USA; Present address: Department of Biology, Boston College, Chestnut Hill, MA USA

**Keywords:** *Strogylocentrotus purpuratus*, Pancreas, Ptf1a, Carboxypeptidase, Pancreatic lipase, Amylase

## Abstract

**Background:**

Digestive cells are present in all metazoans and provide the energy necessary for the whole organism. Pancreatic exocrine cells are a unique vertebrate cell type involved in extracellular digestion of a wide range of nutrients. Although the organization and regulation of this cell type is intensively studied in vertebrates, its evolutionary history is still unknown. In order to understand which are the elements that define the pancreatic exocrine phenotype, we have analyzed the expression of genes that contribute to specification and function of this cell-type in an early branching deuterostome, the sea urchin *Strongylocentrotus purpuratus.*

**Results:**

We defined the spatial and temporal expression of sea urchin orthologs of pancreatic exocrine genes and described a unique population of cells clustered in the upper stomach of the sea urchin embryo where exocrine markers are co-expressed. We used a combination of perturbation analysis, drug and feeding experiments and found that in these cells of the sea urchin embryo gene expression and gene regulatory interactions resemble that of bona fide pancreatic exocrine cells. We show that the sea urchin *Ptf1a*, a key transcriptional activator of digestive enzymes in pancreatic exocrine cells, can substitute for its vertebrate ortholog in activating downstream genes.

**Conclusions:**

Collectively, our study is the first to show with molecular tools that defining features of a vertebrate cell-type, the pancreatic exocrine cell, are shared by a non-vertebrate deuterostome. Our results indicate that the functional cell-type unit of the vertebrate pancreas may evolutionarily predate the emergence of the pancreas as a discrete organ. From an evolutionary perspective, these results encourage to further explore the homologs of other vertebrate cell-types in traditional or newly emerging deuterostome systems.

**Electronic supplementary material:**

The online version of this article (doi:10.1186/s12862-016-0686-0) contains supplementary material, which is available to authorized users.

## Background

The ability of a species to survive in a new environment is strictly related to its capacity to successfully digest and assimilate different food sources. A characteristic that is shared by all the living forms is the ability to digest large molecules available in their habitat to produce energy. Digestion relies on the function of enzymes that are able to break down proteins, lipids and sugars in the diet. The number of specialized enzyme-producing cell types involved in food digestion increases with the complexity of the animal body and the variety of the meal. Metazoans typically have specialized exocrine cell types for the secretion of digestive enzymes. For example, gland cells that produce and release zymogens in the gastric cavity are scattered along the hydra gastroepidermis [1, 2] and along the digestive filaments of corals [3]. Cells rich in granular inclusions involved in extracellular digestion are localized along the foregut and in the gut caeca of different species of flat worms [4–6]. In different regions of the *Drosophila* midgut there are enterocytes that secrete a vast array of digestive enzymes, with expression levels matching organismal requirements [7]. A specialized organ geared toward the production of digestive enzymes and hormones, the pancreas, appeared uniquely in the vertebrate lineage. The pancreas is a complex organ evolved mainly for food digestion (exocrine cells) and maintaining blood sugar levels (endocrine cells). The exocrine pancreas constitutes the majority of the mass of the pancreas and it is composed by exocrine cells grouped into acini that secrete enzymes into the intestine [8]. The pancreas originates early in development from progenitor cells located within the endodermal epithelium. Two members of the basic helix-loop-helix (bHLH) family of protein are known to be critical for the exocrine pancreas differentiation and identity, Ptf1a (pancreatic transcriptional factor 1) and Mist1 [9, 10]. Ptf1a has a double role, first in early pancreas specification, and later in acinar cell differentiation and maintenance [11–13], while Mist1 is necessary for full maturation of the acinar cell phenotype [14]. In the mouse, it has been found that in the absence of Hnf1β, *Ptf1a* expression is not induced, leading to defective specification and reduction of the ventral and dorsal pancreas [15]. Furthermore, several signaling factors are involved in balancing exocrine cell proliferation and differentiation. Among all, Notch appears to prevent pancreatic exocrine development, while FGF signaling mediates growth, morphogenesis and differentiation of exocrine cells [16–18]. In both mouse and zebrafish, Notch and its target genes inhibit the activity of the Ptf1 complex in the exocrine cells, and loss of Notch function results in accelerated development of exocrine pancreas [19].

One of the most abundant and evolutionary conserved miRNA involved in pancreas development is miR-375. MiR-375 negatively regulates glucose-stimulated insulin exocytosis by targeting myothrophin mRNA, a protein involved in insulin secrection in pancreatic β-cells [20]. A loss of function approach in zebrafish revealed that miR-375 is essential for the formation of insulin producing β-cells because its knockdown mainly results in malformation of the endocrine pancreas [21]. In addition, studies on mice lacking miR-375 demonstrated that it controls β- and α-cells mass by regulating genes involved in cellular growth and differentiation [22].

Echinoderms—a group of animals that comprise sea urchins, sea stars, sea lilies, brittle stars and sea cucumbers- belong to the deuterostome clade. The relatively simple development of sea urchin embryos, coupled with the availability of molecular tools for gene perturbation, enable comparative studies on cell specification and developmental mechanisms with other deuterostomes [23]. In particular, echinoderm systems present, in the comparison with vertebrate deuterostomes, the great advantage of a simplified gene toolkit, since this group split before the duplication events occurred at the emergence of Craniata. Importantly for this study, *Strongylocentrotus purpuratus* has an indirect development that generates a bilateral free-swimming larva whose behavior is centered on feeding.

The sea urchin larval gut is a simple tripartite tract composed of a muscular esophagus that exhibits strong contractions, a large spherical stomach with sphincters at both openings, and a small tubular intestine, which exits through the anus. In the stomach of sea urchin larvae, there are cells with the morphological features of zymogen secreting cells that are able to synthesize digestive enzymes, such as β-glucanase and α-amylase [24–27]. Gastric exocrine cells have been described also in the stomach of the adult form of *S. purpuratus*. These cells are highly concentrated in the upper stomach and electron microscopy analyses reveal that they are strikingly similar to mammalian zymogen cells, such as cells of the gastric mucosa and pancreatic acinar cells [28].

Although pancreatic cell types have been extensively characterized in the main vertebrate model systems, most of the studies in non-vertebrate animals have examined gut cells at the morphological level only and molecular information is still very scarce. Therefore, a few data are currently available on the genes/genetic characterization of the pancreatic exocrine-like cell types in non-vertebrate metazoans. In this study, we use a candidate gene approach to identify and characterize the pancreatic exocrine cell type in the sea urchin embryo and larva. We defined a unique population of cells clustered in the sea urchin larva upper stomach that responds to food and expresses the homologs of the pancreatic genes *Ptf1a* and *Mist1* together with at least three digestive enzymes. Our findings are a first step to understand the evolution of pancreatic exocrine cells. We report that the components of the specification pathway and expression for zymogen genes are active in an early branching deuterostome, which suggests that the exocrine cell type may predate the evolution of the vertebrate pancreas.

## Methods

### Animal husbandry, embryo and larva cultures, feeding experiments

Adult *S. purpuratus* were obtained from Patrick Leahy (Kerchoff Marine Laboratory, California Institute of Technology, Pasadena, CA, USA) and housed in circulating seawater aquaria at the Stazione Zoologica Anton Dohrn of Naples. Adult sea urchin maintenance, embryo and larva cultures and feeding experiments were performed as previously described [29].

### RNA *whole mount in situ hybridization*

For fluorescent whole mount *in situ* hybridization (FISH), we followed the protocol outlined in Cole et al. 2009 with the modification described in [30]. Signal was developed with fluorophore-conjugated tyramide (1:400 reagent diluents, Perkin Elmer) following instructions. For all the genes, labeled probes were transcribed from linearized DNA as described in [31]. SpmiR-375 probe has been synthesized and DIG labelled from Exiqon, and the sequence is: 5’/DigN/TGACGCGAGCCGAACGAACAAA/3’DigN/. The double FISH procedure for SpCpa2L and SpmiR-375 was performed as cited with the only exception that the miRNA probe concentration was 0025 pmol/μl and the samples were hybridized 5 days at 42 °C. mir375 probe has already been used by Christodoulou et al. 2010. Primers used to amplify the riboprobes are summarized in Additional file 1. Templates of all the probes were sequenced prior to probe generation and cloned in the pGEM®-T Easy Vector (Promega, Madison, WI, USA). Sense probes were synthesized to test the specificity of the antisense probe signal. FISH was imaged with a Zeiss 510 Meta confocal microscope.

### DAPT treatment

The γ-secretase inhibitor DAPT (N-[N-(3,5-difluorophenacetyl)-L-alanyl]-S-phenylglycine t-butyl ester, Sigma–Aldrich, St. Louis, MO) [32] was dissolved in DMSO and added at 17 h to a final concentration of 8 μM, to avoid the early and toxic effects of the drug, as described in Materna et al. 2012. A corresponding volume of DMSO was added as control.

### Perturbation experiments with MO injection

For each experiment and for each morpholino oligonucleotide (MO), 200 to 400 eggs were injected with approximately 2-4pl of oligonucleotide injection solution and each experiment was repeated three times. As a negative control, fertilized eggs were injected with the standard control morpholino (GeneTools, Philomath, OR) and compared side-by-side with knockdown embryos. The injection of the standard control morpholino did not have any effect on the development of embryos. In a pilot experiment, different concentrations of the MASOs were injected and the morphant’s phenotype was observed in order to test for the right concentration and avoid secondary effects or MASO toxicity. The highest concentration that was not toxic for the embryos was used in each experiment. The injected embryos were cultured at 15 °C until 50 h and 67 h of development, when they were fixed and used to analyze the expression of target genes in FISH or qPCR experiments. A translation-blocking antisense MO against SpHnf1a was used at a final concentration in the injection solution of 200 μM (5’-CTAGTTCGTCACCCGAATGCAGCAT-3’, first tested by Peterson and Davidson 2011). A translation-blocking antisense MO against SpPtf1a was newly designed and used at a final concentration of 300 μM (SpPtf1a_MO1: 5’-ATATTTTCCATAGTGATCTCTGAGT-3’). A second translation-blocking antisense MO against SpPtf1a was injected at a final concentration of 150 μM and used to confirm the specific effect of SpPtf1a MO_1 (SpPtf1a_MO2: 5’-GCGCAGGTGGATTATCAAATTGTTC-3’).

### RNA extraction and quantitative real-time PCR (qPCR)

For qPCR analysis of temporal expression profiles, total RNA was isolated from cultures derived from three mixed batches and extracted with Eurozol (EuroClone, Celbio, Milan, Italy). For SpPtf1a morphants and relative controls and for feeding experiments, RNA was extracted using the RNAquous kit (Ambion). For all the experiments, samples were treated with DNase I (Ambion) to remove DNA contamination as described by the manufacturer. First-strand cDNA was synthesized from total RNA using the SuperScript VILO™ cDNA Synthesis Kit (Invitrogen) following the manufacturer’s instructions. Reactions were performed as described in [33] using the ViiA7 REAL TIME PCR with SYBR Green chemistry (Applied Biosystems, Foster City, CA). For all qPCR experiments, ubiquitin mRNA was used as a reference because remains relatively constant during development [34]. For knockdown and feeding experiments, three biological replicates were analyzed, ddCt values were calculated between experiment and control embryos and converted to fold differences using ubiquitin as a reference (FD = 2 ^ΔΔCt^). A threshold of 2-fold difference was chosen as a significant change. Primer sets were chosen to amplify products 100 to 200 bp in length and sequences are reported in Additional file 1. qPCR experiments in different feeding conditions and the relative statistics were performed as previously described in Perillo and Arnone 2014.

### Luciferase assay

The coding sequences of E47, Rbpl, rnPtf1a and spPtf1a were cloned into the pCDNA3.1 backbone. Ptf1a Luciferase assay was conducted as previous described [35]. Briefly, 293 t or Hela cells were plated at a density of 1x10^5^ cells/well in a 24-well plate and transfected with 512.5 ng total DNA/well using Fugene 6 transfection reagent (Promega). For individual constructs, the following concentrations were used (per well): 12.5 ng renilla (pRL-TK, Promega), 125 ng 4x ptf1a:luciferase reporter (contains 4x Ptf1a binding motif cloned from rat *Chymotrypsinogen B* promoter, gift of Dr. Masashi Kawaichi), 125 ng human E47, 125 ng Rbpl and 125 ng of either rat rnPtf1a or sea urchin spPtf1a. Different combinations of plasmids were cotransfected and pcDNA3.1 (Invitrogen) was used as filler DNA to make the total amount of DNA per well uniform across different conditions. Two days post-transfection, cells were harvested and analyzed for firefly and renilla luciferase activities using the Dual-luciferase reporter assay system (Promega) per manufactor’s protocol.

### Bioinformatics analysis

To identify putative Ptf1a binding sites on sea urchin genes of interest, we used the list of Ptf1a binding sites of the mouse acinar digestive enzyme gene promoters published by Masui et. al. (2007). From this list we built base frequency tables for the E-box and TC-box separately, i.e. counts the number of times each base occurs at each position. The base frequency table has four rows (one row for each letter: A, C, G and T) and the number of columns is equal to the motif length. Then, we calculated the weight matrices for the E-box and TC-box, the matrices have the same number of rows and columns as the corresponding frequency tables, with the value at each position being the natural logarithm of the value from the frequency table divided by the number of sequences, i.e. the weight matrices contain the estimates of the log-probabilities of each base occurring at each position in true binding sites, based on the sample of known sites. Since the gap between the two boxes is about one or two helical DNA-turns, we introduced a penalty function based on a cosine function, whose period and phase shift are 10.5 and 21 base pairs separately. Thus for a given sequence, we can calculate a matching score based on the sum of the weights that correspond to the sequence, which should be equal to the log-probability of seeing that sequence given that it is a binding site, and also the gap penalty function for the distance between the boxes.

## Results

### Characterization of regulatory and differentiation signatures for sea urchin pancreatic exocrine-like cells

We identified sea urchin orthologs of regulatory genes with a known conserved role in pancreatic exocrine cell development. The embryonic temporal expression of the transcriptional factors (TFs) analyzed in this study agrees with data already published from other authors [36, 37], but our analysis includes also late larval stages.

**Fig. 1:**
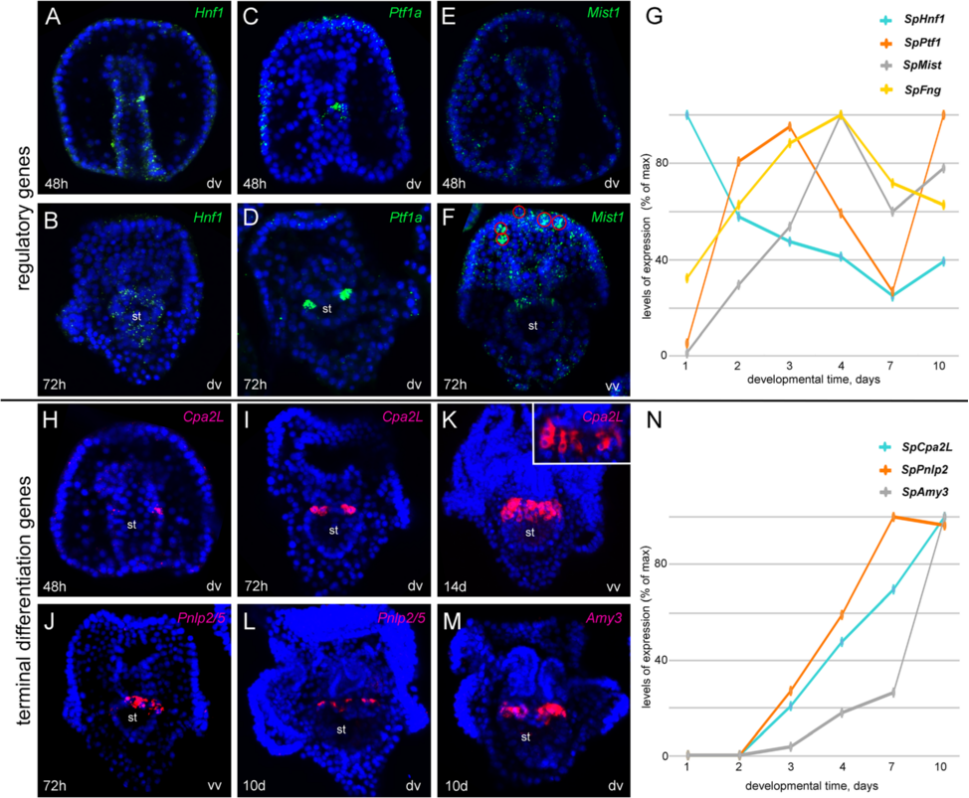
Expression analysis of regulatory and terminal differentiation orthologs of known pancreatic genes. mRNA localization of sea urchin regulatory (**a**-**f**, *green*) and terminal differentiation (**h**-**m**, magenta) genes. For all the figures in this paper, every picture is a full projection of merged confocal Z stacks and nuclei are stained with DAPI and depicted in blue. Red circles in **f** show *Mist1* RNA localization in alternating cells of the apical organ. Inset in panel **k** is a representative single confocal section of the upper stomach of a late larva showing that *SpCpa2L* transcripts are abundantly expressed in the entire cell. **g**, **n** Temporal expression profiles of pancreatic regulatory and terminal differentiation genes during sea urchin development. The graphs show the relative transcript abundance normalized against ubiquitin mRNA. The results are expressed as percentage of the maximum value, corresponding to the stage with the highest level of expression. Standard deviations of three technical replicas are all <0.5. For the sake of simplicity, for each panel, the species in the gene name has been omitted. Abbreviations: d, days; dv, dorsal view; h, hours post fertilization; vv, ventral view

The hepatocyte nuclear factor (HNF) gene family is part of the HNF homeobox class and includes HNF1α and HNF1β, two paralogs with known interchangeable functions in several contexts [38, 39]. The sea urchin genome has only one homolog named *SpHnf1*. Its expression begins in the presumptive endoderm at blastula stage and by mid-gastrula appears confined to the gut [37]. To clarify its later functions in development, we defined the spatial expression of *SpHnf1* in post-gastrula stage embryos. During gastrulation, *SpHnf1* is expressed throughout the hindgut and the midgut, but particularly enriched in a group of cells just below the cardiac sphincters (Fig. 1a). This expression fades away in the early pluteus (Fig. 1b) and it is not detectable in older stages, consistently with the low quantitative level of expression exhibited by this gene at larval stages (Fig. 1g).

The sea urchin ortholog of *Ptf1a* [36] has two distinct domains of expression. At gastrula stage (48hpf, Fig. 1c), transcripts start to accumulate in cells situated right-lateral toward the dorsal side of the midgut, next to the cardiac sphincter, and some hours later it equally expands on both sides. The second domain of expression is in the ectoderm, particularly at the apical organ and in scattered cells of the ciliary band. Throughout the larval stages, the stomach cells increase in number, and we also observe an increase in *SpPtf1a* positive cells (Fig. 1d). The expression of another bHLH gene, *SpMist1* [36], starts at late gastrula stage in the upper stomach and in scattered cells of the apical organ and ciliary band (Fig. 1e, f). *SpMist1* gene expression increases during gastrulation (2 days), although at early pluteus stage (3 days) *SpPtf1a* decreases while *SpMist1* reaches its maximum expression (Fig. 1g). Similarly, the expression of both genes decreases at 7 days after fertilization and increases again after a few days.

We next identified the genes that most likely encode for enzymes used in food digestion. We compared the predicted ORFs of digestive enzyme gene families with the ESTs of an annotated sea urchin late larva cDNA library (MPMGp691, http://owww.molgen.mpg.de/ag_seaurchin/, [40]). The carboxypeptidase family was the first to be analyzed. Among 7 sea urchin orthologs, only SpCpa2L showed a significant representation in the above mentioned larval cDNA library. As shown in Fig. 1h*, SpCpa2L* transcripts are expressed at gastrula stage in two groups of cells in the upper midgut, next to the cardiac sphincter. In a temporal progression, *SpCpa2L* expression begins at the upper lateral stomach cells and later it extends ventrally from both sides until coalescing at the ventral most cells, while leaving a gap at the dorsal most part of the stomach (Fig. 1i and k). Based on the EST abundance at larval stages, *SpPnlp2* and *SpPnlp5* were selected among the lipases and blast analysis revealed that they encode the same protein, hereby named *SpPnlp2/5.* Likewise, *SpAmy3* was selected among the amylases*.* For both genes, transcripts are detectable one day after *SpCpa2L* starts to be expressed, and they are produced in the same above described cells of the upper stomach (Fig. 1j-m). Consistently, the levels of expression of these three genes steadily increase during development and it reaches a peak at 10 days (Fig. 1n).

### Pancreatic exocrine-like cells are clustered in the sea urchin larva upper stomach

**Fig. 2:**
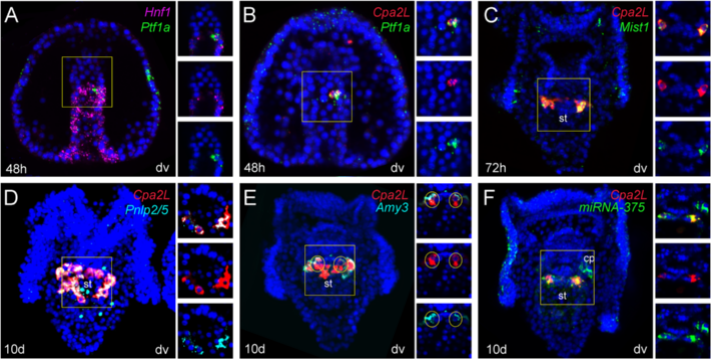
Co-expression analysis of markers of pancreatic exocrine cell-types. Double FISH of selected pancreatic genes in the sea urchin embryos and larva (dv). On the right of each panel, split and combined channels of single confocal sections of the gut domain expressing the two genes (the region of the embryo shown in the right insets is *underlined by a yellow square *) are provided to confirm that the two genes are indeed expressed in the same cells

Our examination of the expression of individual pancreatic regulatory and terminal differentiation genes suggests that they are expressed within a common exocrine-like cell population. To further test this premise, we tested co-expression of these factors by double fluorescent in situ. The endodermal expression of *SpPtf1a* is initially confined to a group of ~3 cells in the midgut within the broad *SpHnf1* domain (Fig. 2a). We next asked if other homologs of pancreatic exocrine genes were co-expressed in those cells. The first digestive enzyme that appears in the *SpPtf1a* and *SpHnf1* positive domain is *SpCpa2L* (Fig. 2b). At gastrula stage, a group of cells in the dorsal-right midgut co-express *SpCpa2L* and *SpPtf1a*. Some hours later, at the pluteus stage, cells expressing these two markers appear ventrally, forming a belt-like structure (Fig. 2c; see also Fig. 2I in [41] for co-expression of these two genes at the pluteus stage). Remarkably, only a few cells of the dorsal stomach do not express these markers. Considering the high expression of *SpCpa2L* transcripts, all confined to the *Ptf1a* positive cells (*SpPtf1a*+), we used this gene expression as a marker for the pancreatic exocrine-like cells. Thus, we analyzed *SpMist*, *SpPnlp2/5* and *SpAmy3* spatial expressions relative to *SpCpa2L* at larval stages. As shown in Fig. 2c-e, all the orthologs of exocrine genes that we considered in this study are co-expressed in the *pancreatic exocrine-like* cells. Remarkably, the same cells express the evolutionary conserved pancreatic microRNA *miRNA-375*. Figure 2f shows that *miRNA-375* is strongly expressed throughout the ciliary band and in the stomach, where it is localized exclusively in the *pancreatic exocrine-like* cells. In addition, this gene is expressed in the coelomic pouches and the probe signal is stronger in the right one.

### Notch signaling inhibits differentiation towards pancreatic exocrine cell fate

**Fig. 3:**
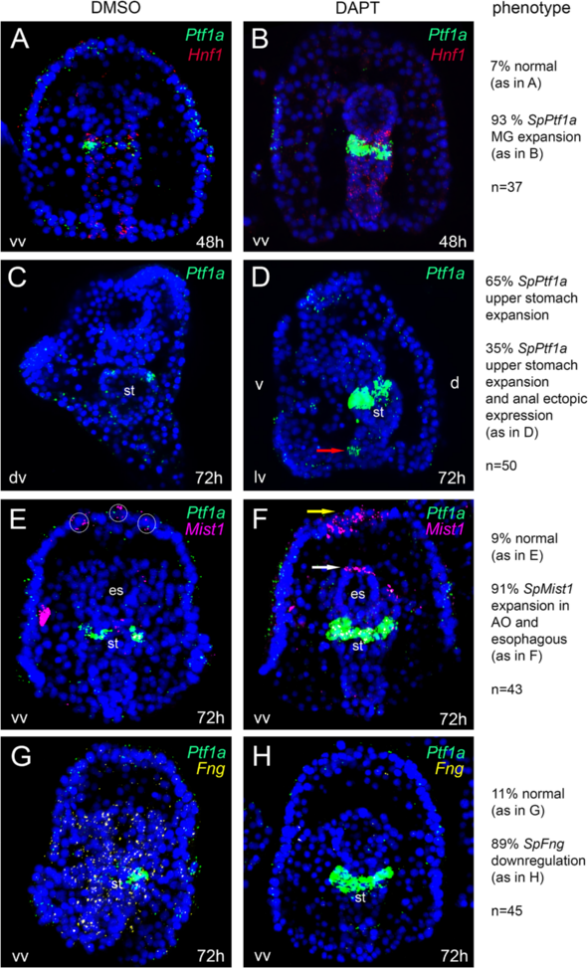
Spatial analysis of gene expression after Notch signaling perturbation. *Hnf1* (**a**, **b**), *Ptf1a* (**c**, **d**), *Mist1* (**e**, **f**) and *Fng* (**g**, **h**) transcript localization tested by FISH in control animals (**a**, **c**, **e**, **g**) and in animals treated with DAPT (**b**, **d**, **f**, **h**). In all the experiments, *Ptf1a* (*in green*) was used as second probe in double FISH (in **a**, **b**, **e**, **f**, **g**, **h**) since the increase of Ptf1a + cells in treated larvae confirms the phenotype. Red arrow in D indicates *Ptf1* ectopic expression in a few cells of the anus. Yellow circles in E show that *Mist1* is expressed in alternated cells of the apical organ in control larvae, while arrows in F indicate that in treated larvae *Mist1* is expressed in adjacent cells of the apical organ (*yellow arrow*) and in a few cells of the esophagus (*white arrow*). For each analyzed gene, quantification of the phenotypes is shown on the right. Abbreviations: AO, apical organ; es, esophagus; lv, lateral view; MG, midgut; st, stomach, v, ventral

Given its known role in pancreatic differentiation, we next tested the role of the Notch signaling pathway in sea urchin pancreatic exocrine-like cell differentiation, using the Notch inhibitor DAPT. The first striking effect of this treatment is the expansion of the *SpPtf1a* + cells in the gastrula and larva upper midgut/stomach (Fig. 3). Notably, the SpPtf1a expansion in the treated embryos never overcomes the cardiac sphincter border. Despite the strong effect on the endodermal cells, ectodermal *SpPtf1a* expression is not affected. Moreover, 35 % of the analyzed plutei showed an ectopic expression of *SpPtf1a* in a small group of cells in the anus (Fig. 3d, *red arrow*). Conversely, the expression of the early endodermal gene *SpHnf1* is not affected by the DAPT treatment at gastrula stage (Fig. 3a, b), in agreement with the previously reported data at blastula stage [42]. When Notch signaling is perturbed, the expression of *SpMist1* exhibits opposite effects than that of *SpPtf1a*. At the pluteus stage, the gene expression in the stomach is unaltered, while in the esophagus and in the apical organ *SpMist1 is turned on also in* cells that are adjacent to the wild type SpMist1+ cells(Fig. 3e and f and *arrows* in Fig. 3f).

Many modulators of Notch signaling are known, among which there is the O-fucose β-1,3 N-acetylglucosaminyltransferase Fringe, whose role is to potentiate Notch activation by Delta [43]. Since a Notch signaling requiring Fringe is involved in specification of endoderm and boundary formation during sea urchin embryonic development [44], we asked if Notch inhibition affects Fringe expression also in the pluteus larva. The sea urchin ortholog of Fringe, *SpFng*, is widely expressed in the pluteus mesoderm, ectoderm and endoderm (Fig. 3g). Our analysis demonstrated that perturbation of Notch signaling causes a consistent reduction of *SpFng* expression in all the tissues where it is normally expressed (Fig. 3h), even in the pluteus.

### Transcriptional control of sea urchin pancreatic exocrine-like cell development

**Fig. 4:**
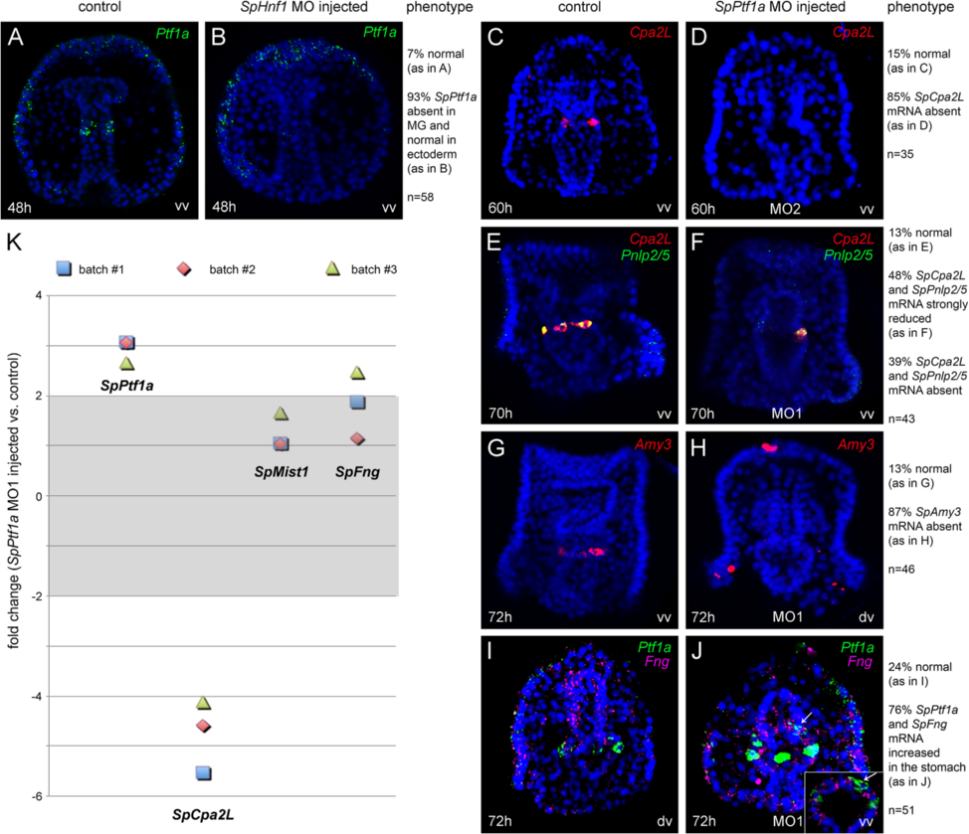
Analysis of gene expression after MO perturbation. *Ptf1a* mRNA localization in control embryos (a) and in embryos injected with MO directed against the translation of SpHnf1 RNA (**b**). *Cpa2L*, *Pnlp2/5*, *Amy3*, *Ptf1a* and *Fng* transcripts were detected by single or double FISH in control larvae (**c**, **e**, **g**, **i**) and in larvae injected with two different MOs directed against the translation of SpPtf1a RNA (**d**, **f**, **h**, **j**). Note that f, **h** and **j** show larvae injected with SpPtf1a MO1. To confirm the effects of MO1, figure **d** shows that SpCpa2L transcripts are absent also in larvae injected with SpPtf1a MO2. Inset in **j** is a representative single confocal section of the dorsal upper stomach of a late larva and the white arrow shows that Ptf1a + and Fng + cells are adjacent and not overlapping. **k** qPCR analysis showing the effects of SpPtf1a MO1 perturbation on transcript levels of selected pancreatic genes at 70 h. Fold changes ≥ -2 and ≤ 2 are shaded in light grey and indicate non-significant changes in gene expression

We next asked if the TFs expressed early in development are involved in a gene regulatory module that specifies pancreatic exocrine cells. To this aim, specific morpholino oligonucleotides were injected in the sea urchin fertilized eggs to block either *SpHnf1* or *SpPtf1a* translation. *SpHnf1* MO sequence specificity has already been demonstrated by Peterson and Davidson (2001), and we used that same morpholino in our experiments. In *SpHnf1* morphants, *SpPtf1a* expression in the midgut is downregulated, while its expression in the ectoderm remains unaltered (Fig. 4a, b). Fluorescent *in situ* hybridization (FISH) or quantitative PCR (qPCR) analyses were performed on larvae to test the effects of SpPtf1a MO perturbation on putative target genes. We observed that the spatial expression of *SpCpa2L*, *SpPnlp2/5* and *SpAmy3* is strongly reduced (Fig. 4e-h). For *SpCpa2L*, which among the enzymes shows the highest transcript expression at 3 days, changes at the transcript levels were also analyzed by qPCR analysis, confirming that its expression is strongly downregulated (Fig. 4k). Conversely, in SpPtf1a morphants additional groups of *SpPtf1a* + cells appear in the upper stomach, both ventrally and dorsally (Fig. 4j) and qPCR data confirm *SpPtf1a* upregulation (Fig. 4k). Additionally, in SpPtf1 morphants there is a modest increase of *SpMist1* expression (Fig. 4k), and a slight increase of *SpFng* transcripts (Fig. 4i, j and k). Particularly, *SpFng* and *SpPtf1* seem to be expressed by adjacent and not overlapping cells (Fig. 4j, insert). To test if the effects of SpPtf1a MO were specific, we injected a second MO to block SpPtf1a translation (SpPtf1a MO_2) and we checked the expression of *SpCpa2L*, since this gene is the first TD marker of this cell type to appear and also the most abundant at pluteus stage. Also in this case, we found that the *SpCpa2L* mRNA is downregulated in the morphants (Fig. 4c and d), confirming that the two morpholinos produce comparable effects.

### SpPtf1a function in mammalian cells

**Fig. 5:**
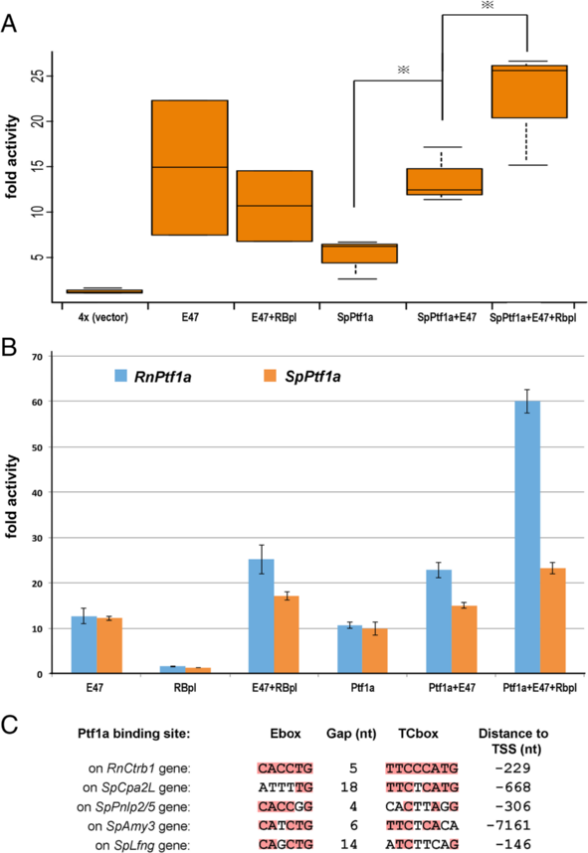
Sea urchin Ptf1a is active and can function together with mammalian Ptf1 partners. **a** 293 t cells were transiently transfected with luciferase reporter containing 4x Ptf1a-responsive element (vector indicated as 4x in the figure) and/or spPtf1a, E47, Rbpl construct; renilla vector was cotransfected for normalization. Results are displayed in the box plot as fold activation over the activity of 4x reporter alone. Transfections were done in triplicate and were repeated 5 times. Luciferase assays from all transfections show similar trend, one representative result is shown. t-test was carried out for group comparison. The bottom and top of the box are the first and third quartiles, and the band inside the box is the median. The whisker represents 1.5 IQR (interquantile range, the differences between the third and first quantile). **b** SpPtf1a has lower activity compared with mammalian Ptf1a in the *in vitro* luciferase assay. Experiment setting is the same as (**a**) except that rat rnPtf1a was transfected in a second plate side-by-side for comparison. We tested SpPtf1a activity in multiple cell lines, one representative result from HeLa cells is shown. **c** Alignment of the closest to the transcription start site (TSS) Ptf1a binding sites found by bioinformatic analysis on *S. purpuratus* (Sp) promoters with the canonical Ptf1a binding site of the RnCtbr1 gene (ref). nt, nucleotides

Ptf1a is part of the PTF1 heterotrimeric complex that also includes one of the common E proteins (E47) and RBPL [45]. To test the ability of the sea urchin Ptf1a protein to substitute for mammalian Ptf1a in this complex, we performed luciferase essays in which either SpPtf1a or rat Ptf1a were co-transfected with mammalian E47 and RBPL, and evaluated for an ability to transactivate a PTF1-responsive luciferase reporter containing four tandem repeats of the PTF1 binding site from the rat chymotrypsinogen promoter (Obata et al., 2001). Figure 5a and b show that, in both 293 t and HeLa cells, SpPtf1a can effectively partner with mammalian PTF1 components to activate the rat chymotrypsinogen element, albeit with lower activity than rat Ptf1a (Fig. 5b). As a correlate of this conserved function, we hypothesized that the sea urchin orthologs of mammalian PTF1a target genes would also display similar PTF1a regulatory motifs. In the promoter of genes encoding mammalian pancreatic digestive enzymes, PTF1 has been found to bind to an extended bipartite DNA sequence that consists of an E-box and a TC-box, spaced one or two helical DNA-turns apart. Masui and coauthors have reported a list of PTF1-binding sites of the acinar digestive enzyme gene promoters for mouse [45]. We used the list of binding sites from that paper and built a position weight matrix for the E-box and TC-box, taking into consideration of the distance between these two elements. This matrix was used to look for Ptf1a binding sites in the promoters of the genes that were affected in SpPtf1a morpholino perturbations. Interestingly, we identified canonical Ptf1a binding sites in sea urchin genes displaying in vivo spPtf1a dependence, including *SpFng*, *SpCpa2L*, *SpPnlp2/5* and *SpAmy3*. An alignment of the documented Ptf1a binding site in the rat *Ctrb1* gene with the putative Ptf1a binding sites in each of the *S. purpuratus* genes is shown in Fig. 5c.

### Pancreatic exocrine-like gene expression response to different feeding regimes

**Fig. 6:**
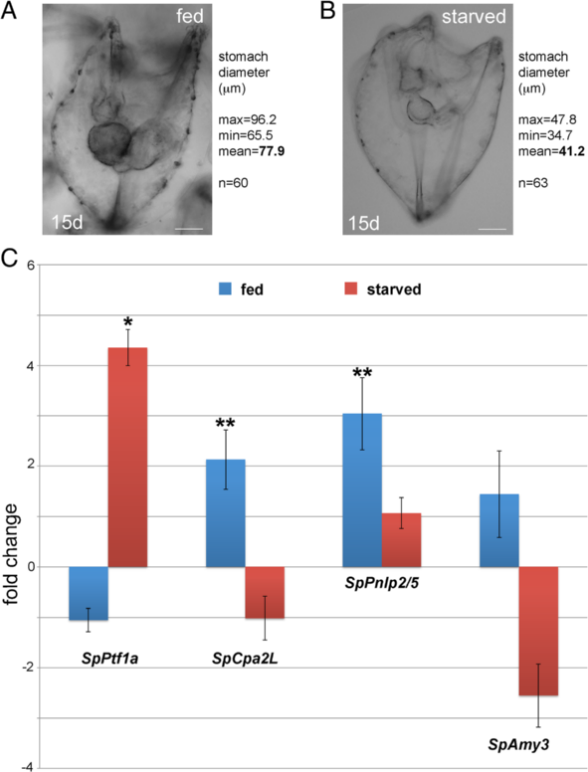
Effect of different feeding regimes on pancreatic exocrine cell gene expression. **a** and (**b**) are bright field images of 15-day larvae that are representative for the fed or starved conditions. Larvae are viewed from lateral right side, mouth up. Stomach diameter was measured from ventral to dorsal, along the midline of the larval body. **c** Quantitative PCR analysis of *SpPtf1a* and digestive enzymes transcripts in feeding and fasting conditions. Data were normalized using ubiquitin as reference gene. Three biological replicas and three technical replicas were measured. The differences between groups that resulted significant by Student’s two tailed t-test are indicated as ** if *p* < 0.01 (highly significant)

We next asked if pancreatic exocrine-like cells respond to different feeding regimes. To this aim, *S. purpuratus* embryos were grown and fed on algae until day 10 to reach the normal larval morphology, then food was removed from the culture media. The culture was split into two batches, of which one was fed for 24 h and the other was fasted for the same time interval. First, we observed larval morphology and found that the different feeding regimes show a clear effect on gut sizes. The stomach and intestine of larvae that were fed is bigger than the ones of the fasted larvae, suggesting that the whole gut epithelium responds to the presence of food (Fig. 6a, b). Second, we compared by qPCR the expression levels of *SpPtf1a* and the previously described digestive enzymes in feeding vs. starving conditions (Fig. 6c). The analysis shows that the expression of the digestive enzymes increases upon feeding. Differently from its putative target genes, *SpPtf1a* expression is higher in fasted larvae than in fed ones. To understand if the increased expression of those genes was due to the same pancreatic exocrine-like cells or to other cells, we tested gene expression through FISH analysis, confirming that the spatial expression of the above genes does not change in feeding or starving conditions (data not shown).

## Discussion

**Fig. 7:**
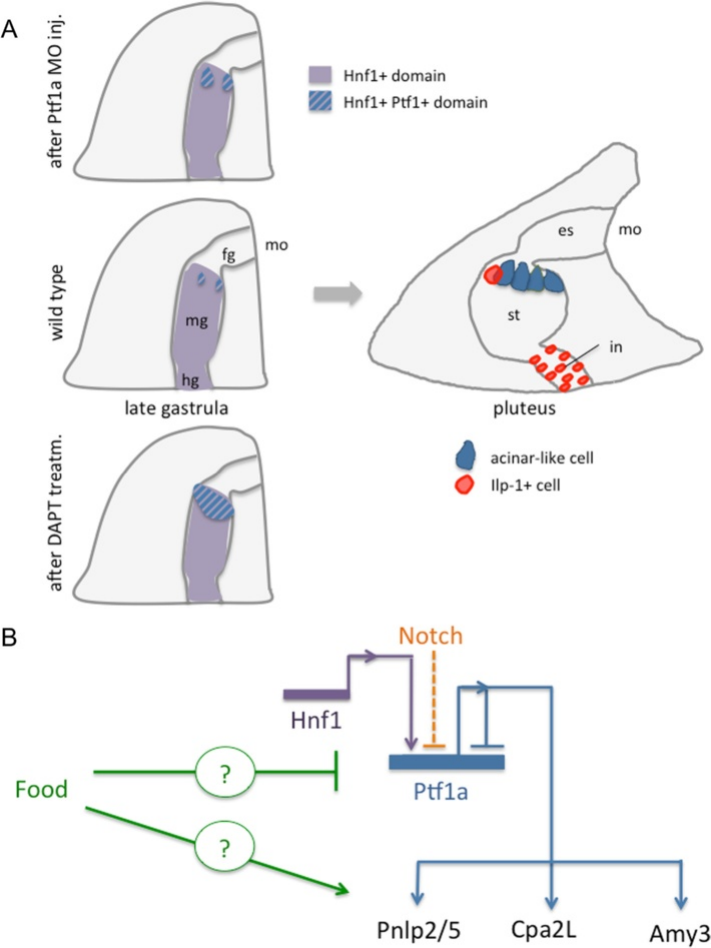
Proposed model of sea urchin pancreatic exocrine-like specification and differentiation. **a** The schemes show the development of exocrine-like cells from late gastrula (*left*) to larva (*right*). The right drawing also shows the localization of putative endocrine cells that are positive for antibody staining of a member of the insulin family (ILP-1+ cells). Note that we do not know if the ILP-1+ cells in the upper stomach are simply adjacent to the pancreatic exocrine-like cells or there are stomach cells which share both features. **b** Summary of regulatory interactions occurring in pancreatic exocrine-like cells unraveled by this study. Different genes are shown with different colors. The wiring among the genes is shown with solid lines. Arrows represent positive regulation, bars represent repression, and dashed lines indicate signaling events. Question marks in the food input means that the mechanism and the genes involved in the pathway are not known. Abbreviations: fg, foregut; hg, hindgut; in, intestine; mo, mouth

In the present study, we show that, in the sea urchin larva, cells specialized in food digestion are clustered in the upper stomach. These cells display a differentiation pathway similar to zymogen cells, where regulative genes (such as *Ptf1a*, *Hnf1* and *Mist1*) and terminal differentiation genes (those encoding digestive enzymes) are co-expressed. Within this upper stomach region, only a few cells on the ventral side do not express the pancreatic markers, denoting a different gene regulatory module and a different function. Figure 7a presents a schematic view of the ontogenetic process leading to the formation of pancreatic exocrine-like clustered cells in the sea urchin embryo and larva. From a temporal point of view, the increase of *SpPtf1a* expression is necessary for the following expression of at least three digestive enzymes that have been analyzed in this study. The expression of these terminal differentiation genes remains constantly high throughout the larval stage, underlying their importance for larval development. *SpPtf1a* and *SpMist1* expression oscillate, and after initial activation, decrease, suggesting that these early TFs may be necessary to activate digestive enzyme transcription but also other factors are involved in maintaining the right level of enzymes during feeding. Interestingly, the pancreatic exocrine-like cells also express the islet-specific microRNA-375. A former study showed that also miR-100, miR-125 and let-7, markers of neurosecretory endocrine cells, are localized in the same area of the sea urchin stomach [46]. These cells seem to be of the pancreatic exocrine-like type, although further analysis will be necessary to test co-expression.

In the developing pancreas, Notch signaling represses endocrine development and exocrine cell differentiation [16, 19, 47]. The early role of Notch signaling in the sea urchin embryos has been extensively studied [48–51]. In this study, we identified a putative recruitment of Notch signaling at the late gastrula stage in blocking the differentiation of stomach cells towards an exocrine-like fate in the pluteus larva. We found that Notch signaling represses *SpPtf1a* expression in those cells that are adjacent to the *SpPtf1a +* cells. Moreover, we observed an ectopic expression of *SpPtf1a* in a group of cells of the anus, suggesting that the Notch signaling could be responsible for the direct or indirect repression of *SpPtf1a* expression in these intestinal cells. Nonetheless, we observed that when Notch signaling is blocked, *SpMist1* ectopically accumulates in the cells of the apical organ that are adjacent to *SpMist1+* cells. One possibility is that a secretory fate is repressed by a Notch lateral-inhibition mechanism in ectodermal cells that are committed to a not-secretory fate. Alternatively, this ectodermal effect may be due to an extra round of cell division. In a similar way, our observations suggest that Notch signaling prevents the exocrine fate of a group of cells of the esophagus. In some cases, we observed that when Notch signaling is blocked, pancreatic genes that are expressed in both the ectoderm and the endoderm lose expression in a specific territory, and not in the other. For instance, the transcription of *SpPtf1a* in the ectoderm and that of *SpMist1* in the stomach do not change in DAPT treated plutei, similarly to what Materna and Davidson observed for *SpShr2* and *SpDelta* in the early embryo [42]. Our data suggest that Notch signaling could have different effects not only during early development, but it can also finely regulate cell differentiation during development in the sea urchin larva. Nonetheless, to deeply analyze the fine mechanisms that underlie regulation of midgut fates, further knockdown experiments of the Notch signaling machinery will be important to enhance our understanding in the future.

The Ptf1a transcriptional complex controls maturation of pancreatic exocrine cells and many efforts has been made to identify its cofactors and targets [52, 53]. In the pancreas, *Hnf1a* activates the transcription of *Ptf1a*, which is part of a transcriptional complex that controls pancreatic exocrine cell maturation and regulates the production of digestive enzymes in acinar cells [13, 54]. Here we found that this gene regulatory module is conserved in an early-branching deuterostome. Based on the homology with mammalian binding sequences, we bioinformatically identified several putative Ptf1a binding sites on the promoters of the sea urchin digestive enzymes which transcription is strongly downregulated in Ptf1a perturbed embryos. We also showed that SpPtf1a can bind mammalian target genes to activate transcription. However, further in vivo experiments are necessary to test the functionality of these binding sites predicted *in silico*. In this study, we also demonstrated that SpPtf1a represses its own gene expression in some stomach cells. We reported that in SpPtf1a morphants, more *SpPtf1a +* cells appear as single pouches at the ventral and dorsal stomach in cells where normally the gene is never expressed. These cells appear as a distinct group of cells that alternate with cells where *SpPtf1a* is absent, like a chessboard (Fig. 4i). Conversely, blocking the Notch pathway results in a continuous ring of Ptf1a + cells (Fig. 4l). Hence, the presence of different mechanisms supports the role of a Notch lateral-inhibition model in the stomach domain. Another finding that emerged from our data is that SpPtf1a may repress the expression of *SpFng*. Due to its role, the Fringe ortholog is widely expressed in the embryos, and this could explain the borderline qPCR values in SpPtf1a morphants. However, our bioinformatic analysis found binding sites for Ptf1a in the *SpFng* promoter region. To understand if the effect of DAPT treatment on SpFng is specific for an exocrine cell fate, further experiments should define whether Ptf1a interacts with corepressor proteins, as in the case of Nkx6 in mammals [55], to block SpFng transcription and consequentially Notch activation, in a negative feedback loop manner.

A summary of the above described putative gene interactions and the proposed gene model leading to specification and differentiation of sea urchin pancreatic exocrine-like cells are schematized in Fig. 7b.

The sea urchin larva is a very plastic organism, able to adapt its metabolic and growth programs to changes in the environmental energy context, e.g. arms and ciliary band growth is influenced by different food availability [56–61]. Feeding has a central role in the life of sea urchin larvae, since they may live up to several months in the plankton, therefore they have to be considered as fully developed animals, even if their body axis will be rearranged after metamorphosis. In a previous study, we found that in the sea urchin larva, a member of the insulin family is expressed in a feeding-dependent fashion, opening new questions on whether other genes are influenced by food assumption or deprivation [29]. As expected during feeding, when more digestive enzymes are necessary, we found that the transcription of a carboxipeptidase, a pancreatic lipase and an amylase are significantly increased, whereas *SpPtf1a* expression showed an opposite behavior. It will be important to further investigate which other factors are involved in the binding of the Ptf1a complex to its targets. Previous transcriptomic analysis reported that *SpCpa2L* and *SpPnlp2/5* transcripts are strongly reduced when *SpLox* is knocked down [62] and it is known that its vertebrate ortholog, *Pdx1*, regulates the development of digestive enzyme-producing acinar cells and insulin expression [63, 64]. In the sea urchin, SpLox is expressed in a group of cells between the lower stomach and the intestine (the pyloric sphincter) [65], in a gut domain and in cells that are different from the herein characterized pancreatic exocrine-like cells. Thus, a possible scenario that could be worth testing in further experiments is that SpLox could act through a hypothetic signaling pathway to eventually regulate the expression of digestive enzymes, accordingly with food availability.

The pancreatic exocrine-like cells that we describe are localized in the same area where there are also endocrine-like cells producing a member of the insulin family, ILP1 [29]. We cannot rule out the possibility that these two cell types are just in close proximity and do not represent a unique cell-type sharing both functions. Generation of antibodies for the digestive enzymes will be necessary to test if these exocrine markers are coexpressed in the ILP+ cells, but we can at least conclude that both cell types are localized in the same area of the stomach. In the cephalocordate amphioxus, endocrine and exocrine cells do not exhibit any gut specialization [8, 66–68]. In the hagfish, an islet-like organ is independent of exocrine acini, and, in the teleost, endocrine islets are scattered among the more widespread exocrine parenchyma [69]. Thus, two possible evolutionary scenarios could be put forward. First, the pancreas and the exocrine and endocrine-like cells (closely localized) in the sea urchin gut are the result of convergent evolution, thus implying that this feature was lost in early branching chordates. Second, the clustering of the sea urchin pancreatic exocrine-like cells could be the result of an independent evolution from an ancestral pancreatic cell-type. To test these hypotheses and to infer the evolutionary origin of the pancreas, homologs of pancreatic genes should be studied in other echinoderms as well as in other non-chordate deuterostomes, such as the hemichordates.

## Conclusions

Here, we report that a gene regulatory module required for vertebrate pancreatic exocrine cell development is shared by a non-chordate deuterostome. Our study, together with previous studies [65, 70], supports the idea that the sea urchin gut is a multifunctional organ and it is composed of highly differentiated cells, which in many aspects are similar to vertebrates, therefore providing an amenable system in which to study development and function of gut cells.

## Additional file

Additional file 1: Supplementary Table 1. (PDF 55 kb)
