# Supplementary material for: A pancreatic exocrine-like cell regulatory circuit operating in the upper stomach of the sea urchin Strongylocentrotus purpuratus larva
Source: BMC Evol Biol. 2016 May 26;16:117. doi: 10.1186/s12862-016-0686-0 (PMC4880809; doi:10.1186/s12862-016-0686-0)
Supplement: Additional file 1: — Supplementary Table 1. (PDF 55 kb) [file 12862_2016_686_MOESM1_ESM.pdf]

Supplementary Table 1

List of primers used for FISH probes

**Gene name**	**Gene ID (echinobase)**	**Forward**	**Reverse**
SpHnf	008196	5-'CCGAATACAGCCCACAAGAT-3'	5'-CGATGGTAGTTTCGTGCTCA-3'
SpPtf1a	015953	5'-GAATGTCTTCGAACCCCTCA-3';	5'-TTACTGTTTGGGTCCCCTTG-3'
SpMist	027623	5'- TGTCTTGGAGCCAGTTCGAC-3'	5'-GCTCGTCATTGAGATCATCG-3'
SpCpa2L	*015178.3*	5'- CCGGACGATAGGTTCTTCAA-3'	5'-TGTTTGCAGAGTGCCTTGAC-3'
SpPnlp2/5	012906<br>015110	5'-CCTGTTGGTATTTTTCCTTACCC-3'	5'-GGTTAACTAGTCCGCCATGC-3'
SpAmy3	014671	5'- AAGATTTTTGGGGCCTTACG-3'	5'-TTGGAGATGTGGGCACAGTA-3'.
SpFng	004621	5'-ATGGCAGGAAAACAAACAGG-3'	5'-GTGGCTGGACAGTTTCCATT-3'

List of primers used for qPCR analysis

**Gene name**	**Forward**	**Reverse**
SpUbi	5'-CACAGGCAAGACCATCACAC-3'	5'-GAGAGAGTGCGACCATCCTC-3'
SpPtf1a	5'-GAATGTCTTCGAACCCCTCA-3'	5'-GATGACATCCGCTTCCTCTC-3'
SpMist	5'-ATGTGAATCTTCGACCGGGT-3'	5'-GGCATCGTTAAGTGTGTGCA-3'
SpHnf	5'-CCGAATACAGCCCACAAGAT-3'	5'-TGCGAGAGATGTGATTGGTT-3'
SpCpa2L	5'- CAGCGATGTGACTGAGATCC-3'	5'-GATCTACTCCTCGGCAAACG-3'
SpPnlp2/5	5'- GACGTCATACACACCGATGC-3'	5'- ATGACTGCATCCCGAGAAGA-3'
SpAmy3	5'-TGGAAATGTTGACGAGGTGA-3'	5'-TTGGAGATGTGGGCACAGTA-3'
SpFng_	5'-GCTGATGAAGTTACTCAGGCAGT-3'	5'-AGCTAGTGCCTTGCTGATGC-3'
